# Supplementary material for: Sub-threshold neuronal activity and the dynamical regime of cerebral cortex
Source: Nat Commun. 2024 Sep 11;15:7958. doi: 10.1038/s41467-024-51390-x (PMC11390892; doi:10.1038/s41467-024-51390-x)
Supplement: Supplementary file 1 — Supplementary Information [file 41467_2024_51390_MOESM1_ESM.pdf]

Supplementary Information for: Sub-threshold neuronal activity and the dynamical regime of cerebral cortex

Oren Amsalem[1], Hidehiko Inagaki[2], Jianing Yu[3], Karel Svoboda[4], and Ran Darshan[5,6,7,8,*]

[1] *Division of Endocrinology, Diabetes and Metabolism, Beth Israel Deaconess Medical Center, Harvard Medical School, Boston, MA, USA*

[2] *Max Planck Florida Institute for Neuroscience, Jupiter, FL, USA*

[3] *School of Life Sciences, Peking University, Beijing, China*

[4] *Allen Institute for Neural Dynamics, Seattle, WA, USA*

[5] *Department of Physiology and Pharmacology, Faculty of Medicine, Tel Aviv University, Tel Aviv, Israel*

[6] *The School of Physics and Astronomy, Tel Aviv University, Tel Aviv, Israel*

[7] *The Sagol School of Neuroscience, Tel Aviv University, Tel Aviv, Israel*

[8] *Janelia Research Campus, Howard Hughes Medical Institute, Ashburn, VA, USA*

Supplementary Note 1. Sub-threshold voltage activity and a model of the vibrissal somatosensory cortex

In the main text we focused on analysis and modeling of the ALM network. Here, we present an analysis of a distinct dataset and develop a network model that suggests a different dynamical regime for layer 4 of the vibrissal somatosensory cortex (vS1), at least under the specific inputs studied, when compared to ALM. Specifically, our modeling and data analysis suggest that, in layer 4 of vS1, the inputs to excitatory neurons are not balanced, whereas for the fast-spiking inhibitory neurons, they are. Consequently, we propose that layer 4 of vS1 operates in a 'partially-balanced' regime, where inhibitory neurons, but not excitatory neurons, are balanced.

Spiking and sub-threshold activity in vS1. Similar to the analysis of ALM neurons, we analyzed the supra- and sub-threshold activity of neurons within the vibrissal somatosensory area (vS1) during the sampling period within a similar decision-making task (Fig.S7A; recorded data from [38, 79]). In each trial, mice used their whiskers to detect the location of a pole, positioned either anteriorly or posteriorly on one side of the head. Mice reported the location of the pole by either licking to receive a water reward (for posterior pole locations) or refraining from licking (for anterior pole locations).

In the delayed-response task we focused on the delay period, in which the mouse movement, like licking, was minimal. Conversely, in the Go/NoGo task, no delay period existed, and it's noteworthy that vS1 activity is influenced by whisking movements [91], particularly when the whisker contacts an object [6, 92]. To mitigate the impact of whisking-related activity during the task, we analyzed vS1 neuron activity during the sampling behavior phase, concentrating on non-whisking intervals (see Fig.S9A-C for a comparison of voltage statistics between whisking and non-whisking periods).

The recorded vS1 data encompassed neurons from both layer 4 and layer 5. Analogous to ALM neurons, neurons within both vS1 layers exhibited high CV_{ISI} values. Their firing rate distributions were well-approximated by a log-normal distribution, with fast-spiking (FS) neurons demonstrating higher firing rates compared to excitatory neurons (Fig.S7B-D, Fig.S8B-D).

When we explored the sub-threshold activity, we found that layer 4 excitatory (L4E) neurons were more hyperpolarized than their layer 5 excitatory (L5E) counterparts (Fig.S7E, Fig.S8E). The distance to threshold for L4E neurons was $21mV \pm 7mV$ (among the $n = 19/37$ cells exhibiting action potentials; threshold was $-39mV \pm 5mV$), while L5E neurons displayed a distance of $17mV \pm 7mV$ (among the $n = 29/38$ cells firing action potentials; threshold of $-35 \pm 5mV$). Consequently, due to their substantial distance from threshold, L4E neurons exhibited exceedingly low spiking rates, with almost all recorded neurons firing solely in response to touch ([38]; Fig.S7B).

Upon comparing the sub-threshold activity of excitatory vS1 neurons with those in ALM, we observed that the resting state of vS1's excitatory neurons (both L4E and L5E) was significantly more hyperpolarized (Fig.S7D top, E and Fig.S8E; unpaired Student t-test, $p < 0.01$). In addition, the voltage fluctuation distributions of vS1's excitatory neurons displayed a tendency to deviate from a Gaussian distribution (illustrated by the L4E neuron distribution in Fig.S7D; average skewness of distribution was 1.0 ± 0.6mV for L4E neurons and 0.9 ± 0.6mV for L5E neurons). Skewness of the distribution and resting potential, especially for L4E neurons, were anti-correlated (Fig.S9E), whereby positively skewed voltage distributions corresponded to neurons farther from the threshold. Consistent with this observation, analysis of the membrane potential trajectories preceding action potential [69] suggested that spikes in L4E neurons were driven by significantly larger depolarizations compared to ALM excitatory neurons (Fig.S9H-I).

Besides the differences in the resting state, sub-threshold activity of excitatory neurons in ALM and vS1 also shared common features. First, as with ALM neurons, there was a large level of heterogeneity in the mean voltage of the neurons (large Δ_V), with some neurons much closer to their spike threshold than others. Second, we found that the level of fluctuations of excitatory neurons in ALM and vS1 was quite similar (Fig.S7E, unpaired Student t-test, $p = 0.278$). Third, both in ALM and vS1 neurons the voltage SDs and means were positively correlated across the population (Fig.1G, Fig.S7G-top, Fig.S8G).

In contrast to the excitatory neurons, the FS neurons in vS1 were situated much closer to their spike threshold and displayed increased voltage fluctuations. These two factors contributed to their higher spike rate. When comparing the FS neurons in vS1 with ALM neurons, we found that the

distributions of mean voltages between the two populations were comparable (Fig.S7E-left, distance to threshold: $12mV \pm 5mV$, unpaired Student t-test, $p = 0.997$, threshold $-37mV \pm 4$ mV). In addition, much like ALM neurons, the distribution of voltage fluctuations in FS neurons was well-represented by a Gaussian distribution (with a skewness of 0.3 ± 0.3mV). Nevertheless, FS neurons exhibited higher levels of voltage fluctuations compared to both the excitatory neurons in vS1 and those in ALM. Moreover, these FS neurons demonstrated a negative correlation between their standard deviations and mean voltages (Fig.S7G-bottom).

A model of layer 4 vS1 network that operates in a partially-balanced regime is consistent with the data. The excitatory neurons in layer 4 of vS1 were quiescent and much more hyperpolarized, with neurons that were around $20mV$ below their spike threshold. This is in contrast to neurons that operate in a fluctuation-driven regime that typically hover close to their spike threshold (Fig.4D). We next sought to investigated the cause for these differences in the resting state of neurons in these two populations.

A majority of the recorded excitatory cells in ALM were layer 5 neurons that are mostly pyramidal, while in layer 4 of vS1 these are spiny stellate cells. Therefore, variations in the distance of the neurons from their spike threshold in vS1 and ALM could potentially result from morphological differences between these two populations. However, although their morphology differs significantly (Fig.3A, Fig.S2C), we found that the estimated average visibility parameter for spiny stellate neurons and pyramidal neurons was not dramatically different. Indeed, the statistics of sub-threshold activity was not very different than the statistics in the ALM network when we simulated the same network of Fig.4A, but with the estimated parameters for spiny stellate cells from Fig.S2C-D instead of layer 5 pyramidal cells (Fig.S11A).

Instead of variations in single neuron parameters and based on the data, we hypothesized that the ALM and vS1 excitatory populations operated in different dynamical regimes that originated from variations in their network connectivity. Specifically, we posit that while ALM operates in a fluctuation-driven regime, characterized by a balance in both excitatory and inhibitory sub-networks, layer 4 of the barrel cortex exhibits a state of *partial balance* (see Methods). Within this regime, the external excitatory currents and the recurrent inhibition within the inhibitory population counterbalance each other, whereas the same balance isn't achieved for the excitatory population. Consequently, excitatory neurons can operate significantly below their spike threshold, while the inhibitory neurons remain close to their spike threshold.

In the case of two populations, reducing the external drive to the excitatory population below a certain value leads to a quiescent excitatory population while maintaining the excitatory-inhibitory balance only for the inhibitory population (Methods, [11, 51]). This is the regime which we term a 'partial-balance' regime. We therefore simulated a network with similar parameters as the ALM network in Fig.4A-C, but reduced the external drive to the excitatory population (Fig.S10A-D, Methods).

Excitatory neurons in the vS1 model were hyperpolarized and unbalanced due to a weak external drive to the excitatory neurons in the model. They did not fire unless they received an additional synchronous external input (Fig.S10B). Their mean voltage was about $20mV$ below their spike threshold (Fig.S10B,C) and, similarly to the data, their voltage mean and SDs were positively correlated (Fig.S10D; here, in contrast to ALM network, the positive mean-SD correlations were due to the reversal potential of the inhibitory synapses and not the neuronal threshold). Interestingly, the sub-threshold fluctuations of the simulated neurons were well-approximated by a Gaussian distribution, and were positively skewed as in the data only when a synchronous external drive was added (Fig.S10E-F).

While the excitatory population were quiescent, the recurrent inhibition and the external feed-forward excitation to the inhibitory population in the model were still approximately balanced; inhibitory neurons fired at a few Hertz, with an average voltage of $-50mV$ (around $10mV$ below threshold; Fig.S10C-D). Thus, with this architecture, the supra- and sub-threshold activity of neurons in the network were consistent with the activity of neurons in layer 4 network of vS1 (compare Fig.S10B-D with Fig.S7E-G). This suggests that, similar to the pyramidal neurons of ALM, uncorrelated spiking activity within layer 4 of vS1 was sufficient to drive the inhibitory neurons to fire. This was not the case for layer 4 excitatory neurons in vS1, which fired due to strong synchronous drive.

Overall, the correspondence between the supra- and sub-threshold voltage activity of neurons in the model and the vS1 data suggests that the vS1 layer 4 network operates in a partially-balanced regime.

Functional implications of a partially-balanced regime What is the computational role of a partially-balanced regime? One possibility is that this regime is advantageous for detection tasks. In this scenario, excitatory neurons that are hyperpolarized will be less sensitive to external and internal fuctuations, which could lead to a reduction in false-positive detection rates. In addition, these neurons may be less affected by weak but synchronous variations in external drive, if inhibitory neurons are balanced. This is because inhibitory neurons in this regime react quickly to external stimuli, as they have many neurons that are ready to fire (a phenomenon known as 'fast tracking'[51]). This ensures that the excitatory population remains far from their spike threshold, unless they receive a strong and synchronous drive. This could be achieved through a 'window of opportunity' based on a delay between the two populations[31, 38]. A partially-balanced operating regime can thus lead to sparse coding and results in early reliable sensory response[93], features that may be suitable for areas involved in stimulus detection such as the somatosensory and auditory cortices. A thorough investigation into the computational roles of this regime is reserved for future research.

The operating regime of layer 5 vS1 network. As we showed, layer 5 excitatory neurons in vS1 had similar firing rates to ALM neurons (a majority of which were also layer 5 neurons), and their mean voltages were closer to their spike threshold than L4E neurons. However, they were still more hyperpolarized than ALM neurons. These observations were consistent with previous reports for layer

2/3 neurons in vS1 cortex [92, 94]. With respect to vS1 layer 4 and ALM models, we note that a clear distinction between a fluctuation- and a non-fluctuation-driven regime (or a fully- and partially-balanced regime) is well-defined only in the theoretical limit of an infinite number of inputs per neuron. In finite networks, in which the balance can only be approximated, the distinction between these two regimes becomes less clear. In this sense, layer 5 neurons in vS1 may operate at the border of these two regimes, with recurrent inhibition only partially balancing the feedforward excitation.

Challenges in interpreting the data. It is important to acknowledge that a stronger excitatory drive to vS1 excitatory neurons in layer 4 could potentially steer the network to operate in a regime similar to that of the ALM network. In fact, increased excitatory input from the thalamus during whisking may be responsible to the depolarization of vS1 excitatory neurons whisking onset [71], and it is known that active touch evokes an overall robust depolarization of excitatory neurons [6, 92]. Thus, it is possible that if the whiskers were engaged in exploring a more intricate object (e.g. [95]), the feedforward input targeting the excitatory layer 4 neurons would increase. This increase could push the excitatory neurons closer to their spike threshold, mirroring the behavior observed in ALM neurons.

Finally, we note that the sample size of the intracellular recordings of inhibitory neurons in vS1 was not large (13 neurons). Yet, the alignment between the firing rates statistics of excitatory and inhibitory neurons in vS1 and the partially-balanced hypothesis, along with analytical predictions and simulations of the sub-threshold statistics of excitatory and inhibitory neurons in vS1 (Fig.S7, Fig.S10), and together with recent evidence on the differences in the distance to threshold of inhibitory and excitatory neurons in vS1 [54] further support our claims and compensates for the challenges of obtaining inhibitory neurons in blind patch experiments in behaving animals.

Supplementary Note 2. Mean-field analysis of sub-threshold statistics of integrate-and-fire neurons: Current-based neurons

In this section we obtain the statistics of the time-averaged voltage and its SD across neurons in the network. We consider the homogeneous case of a current-based integrate-and-fire neuron ($\Gamma = \rho = 0$, and we absorb α into g_{ab}). We assume that the network is asynchronous and follow standard mean-field approach to describe the total input to a neuron, $I^a_{tot,i}(t) = I^a_{l,i}(t) + I^a_{rec,i}(t) + I^a_{ext,i}(t)$, by a Gaussian process:

$$I^a_{tot,i}(t) \approx \mu^a_i + \sigma_a \eta^a_i(t) \tag{16}$$

where μ^a_i is the time-average input, σ_a is the SD and $\eta^a_i(t)$ is a white-noise term (assuming $\tau_{syn} \ll C/g_l$, [13]). The input SD, σ_a, is the same for all neurons in the population (see below). In contrast, the mean input, μ^a_i, is different across neurons due to variations in the pre-synaptic spike rates and the number of pre-synaptic inputs per neuron. In large networks the statistics of this time-average input, which determines the heterogeneity of the mean input currents across the population, is Gaussian. We

thus write $\mu_i^a = \mu_a + q_a z_i^a$, with random variables, z_i^a, that are drawn independently from a Gaussian distribution with mean 0 and variance 1.

The total input to a neuron of population a under this mean-field analysis is thus fully described by three sufficient statistics, $s_a = \{\mu_a, q_a, \sigma_a\}$. With this input statistics, the voltage dynamics of a neuron with the inputs of Eq.(16) is reflecting a realization of a typical neuron in the network. Inserting Eq.(16) into Eq.(13) leads to a Langevin equation. The voltage dynamics is thus given by a Fokker-Plank (FP) equation, from which it is possible to calculate the transfer function that relates the mean input and its fluctuations to the mean firing rate [13, 19]:

$$\nu_{ia} = \phi_\nu(\mu_i^a, \sigma_a) = \tau^{-1}\sqrt{\pi}\int_{V_{min}}^{V_{max}} dwe^{w^2} erfc(-w)]^{-1} \tag{17}$$

with $\tau = C/g_l$, $V_{min} = ((V_r - \mu_i^a)/g_l - V_l)/\sigma_a$ and $V_{max} = ((V_{th} - \mu_i^a)/g_l - V_l)/\sigma_a$.

Using the equilibrium distribution of the FP equation, one can also calculate the moments of the voltage distribution [96]. For example, the time-average voltage of a neuron (i, a) is:

$$\bar{V}_{ia} = \phi_{\bar{V}}(\mu_i^a, \sigma_a) = V_{pas}(\mu_i^a, \sigma_a) - \tau\phi_\nu(\mu_i^a, \sigma_a)V_- \tag{18}$$

with $V_- = V_{th} - V_r$ and $V_{pas}(\mu_i^a, \sigma_a) = V_l + \frac{\mu_i^a}{g_l}$. Similarly, the voltage SD yields:

$$\sigma_{V,ia}^2 = \phi_{\sigma_V^2}(\mu_i^a, \sigma_a) = \sigma_{pas,a}^2(\sigma_a) - V_-\tau\phi_\nu(\mu_i^a, \sigma_a)(\phi_V(\mu_i^a, \sigma_a) - V_+) \tag{19}$$

with $V_+ = \frac{V_{th}+V_r}{2}$ and $\sigma_{pas,a}^2 = \frac{\tau\sigma_a^2}{2C^2}$.

The equations for the mean and SD of the voltage are simple to understand. The first term in Eq.(18) and Eq.(19) results from bombarding a passive neuron with Poissonian inputs. The second term in these equations results from the spiking mechanism and mainly affects neurons that are close to threshold. It is proportional to the rate of the events $(\tau\nu)$ and the size of the reset, V_-.

Using Eqs.(17)-(19) we can then calculate the mean and the variance of the voltage and rate distributions across the population by averaging over the neurons:

$$\begin{aligned} X_a &= \int Dz\phi_X(\mu_a(z), \sigma_a) \\ \Delta X_a^2 &= \int Dz\delta\phi_X^2(\mu_a(z), \sigma_a) \end{aligned} \tag{20}$$

with $X \in \{\nu, \bar{V}, \sigma_V\}$ and $\delta\phi_X = \phi_X - X_a$, $\mu_a(z) = \mu_a + q_a z$, $z \in \mathcal{N}(0, 1)$, $Dz = \frac{e^{-z^2/2}dz}{\sqrt{2\pi}}$. For example, voltage heterogeneity is calculated as:

$$\Delta_V^2 \equiv \Delta\bar{V}_a^2 = \int Dz\delta\phi_{\bar{V}}(\mu_a(z), \sigma_a) \tag{21}$$

Importantly, in a regime in which excitation and inhibition currents are canceling each other, both the mean current, μ_α, as well as the disorder across the neurons, q_α and the time-dependent, σ_α are all on the same order of magnitude. As a result, voltage heterogeneity in current-based spiking networks is finite.

Correlations between voltage mean and SD across neurons in the network. Both in ALM and vS1 data, as well as in our network simulations, we observed that the voltage mean and SDs of neurons can be positively or negatively correlated, depending on the network state. We use the mean field analysis for $\Gamma = 0$ to explain this correlation structure.

Equation (19) shows that for neurons that are far from threshold, the voltage SD is governed by the term $\sigma^2_{pas,a}$, which only depends on the input fluctuations, σ_a. Thus, fluctuations of these neurons are the same, independent of their mean voltage. When the mean input current is sufficiently large, both the mean voltage and the SD saturate (due to the threshold). Thus, we expect that the input noise (σ_a) will determine the correlations strength (and its sign) between the voltage SD and its mean.

To show the dependence of the mean-SD correlation on the input SD (or similarly, the voltage SD), we fix the mean rates and SDs of the neurons to be in the range of the pyramidal neurons in ALM ($6 \pm 8Hz$), and numerically invert Eqs.(20) with $X = \nu$, while changing σ_a to obtain μ_a and q_a. We then use the sufficient statistics, $s_a = \{\mu_a, q_a, \sigma_a\}$, to plot the correlations between the mean and voltage SD:

$$Corr(\sigma_a) = \frac{\int Dz\delta\phi_V(\mu_a(z),\sigma_a)\delta\phi_{\sigma_V}(\mu_a(z),\sigma_a)}{\int Dz'\delta\phi^2_V(\mu_a(z'),\sigma_a)\int Dz''\delta\phi^2_{\sigma_V}(\mu_a(z''),\sigma_a)} \tag{22}$$

against the average voltage SD (Fig.4J, left), given by

$$\sigma^2_V = \int Dz\phi_{\sigma^2_V}(\mu_a(z),\sigma_a) \tag{23}$$

This analysis shows that correlations in the mean-SD voltage across the population is determined by the level of the input SD.

Finally, the inhibitory neurons in our network simulations operate in a regime in which they fire at higher rates than the excitatory neurons. This, together with the strong recurrent interactions to the inhibitory neurons, lead to higher input SD for the inhibitory neurons than for the excitatory neurons in the network (see parameters). As a result, the mean and voltage SDs of the excitatory neurons are positively correlated, while they are less, and even negatively, correlated for the inhibitory population (Fig.4E,J).

Supplementary Note 3. Mean-field analysis of sub-threshold statistics of integrate-and-fire neurons: Conductance-based neurons

Here we obtain the statistics of the time-averaged voltage for the homogeneous case of a conductance-based integrate-and-fire neuron ($\Gamma = \rho = 1$, and we absorb α into g_{ab}). In contrast to the case of current-based neurons, for which we only need $(\mu_a.q_a, \sigma_a)$ to describe the distributions of the input to the neurons and thus the rate and voltage distributions of the population, in the case of conductance-based synapses we need six sufficient statistics, $s_a = \{\mu_{aE}, q_{aE}, \sigma_{aE}, \mu_{aI}, q_{aI}, \sigma_{aI}\}$. These are the statistics that define the Gaussian input of the total excitatory and total inhibitory synaptic conductances to a neuron

(i, a):

$$\sum_j g_{ij}^{aE} s_j^E(t) + (\sqrt{K}\bar{I}_{ext,a} + q_{ext,a} z_i^{ext}) \approx \mu_i^{aE} + \sigma_{aE}\eta_i^E(t) \tag{24}$$

$$\sum_j g_{ij}^{aI} s_j^I(t) \approx \mu_i^{aI} + \sigma_{aI}\eta_i^I(t)$$

with $\mu_i^{ab} = \mu_{ab} + q_{ab} z_i^b$, $z_i^b \sim \mathcal{N}(0,1)$ and σ_{ab}^2 is the variance of the white noise term η_i^a, with $\langle \eta_i^a(t)\eta_j^b(t')\rangle = \delta_{ij}\delta_{ab}\delta(t-t')$. For simplicity of notations we write the statistics in vector notations, $s_a = \{\boldsymbol{\mu}_a, \boldsymbol{q}_a, \boldsymbol{\sigma}_a\}$ and $\boldsymbol{\mu}_i^a$.

Similarly to the case of current based neurons, it is possible to use a Fokker-Planck (FP) formalism to analytically describe the firing rate and membrane potential distribution of a conductance-based neuron [40]. We can then use the equilibrium FP distribution to calculate the moments of the distribution (see below). For example, the time-average voltage of a neuron (i, a) yields:

$$\bar{V}_{ia} = V_{pas,i}(\boldsymbol{\mu}_i^a) - V_{-}\tau_{pas,i}(\boldsymbol{\mu}_i^a)\nu_i(\boldsymbol{\mu}_i^a, \boldsymbol{\sigma}_a) \tag{25}$$

with

$$\tau_{pas,i} = \frac{\tau}{1 + \tau(\mu_i^{aE} + \mu_i^{aI})} \tag{26}$$

$$V_{pas,i} = \tau_{pas,i}(V_l/\tau + \mu_i^{aE} E_E + \mu_i^{aI} E_I)) \tag{27}$$

Thus, the mean of the voltage for conductance-based integrate-and-fire neurons have the same form as the mean voltage of the current-based integrate-and-fire neurons (Eq.(25)). It consists of the mean of a passive neuron, bombarded by Poissonian inputs, together with another term that results from the threshold.

Voltage heterogeneity in one compartment models Equations (25)-(27) explain why the amount of voltage heterogeneity in conductance-based networks is low for large networks. Unlike networks of current-based integrate-and-fire neurons, which have currents that can be dynamically balanced to have μ_a and q_a both be $\mathcal{O}(1)$ [51], conductance-based neurons have no balancing of the conductances [41]. This leads to an average conductance, $\boldsymbol{\mu}_a$, that dominates the quenched disorder, $\boldsymbol{q}_a$, which is always a $\sqrt{K}$ order of magnitude smaller than $\boldsymbol{\mu}_a$ due to the central limit theorem . Therefore, the distribution of mean voltages is very narrow in conductance-based networks of one compartment.

To analyze this more systematically, we will focus on the voltage heterogeneity in a passive neuron (Eq.(27)) and will write down the average conductance and quenched disorder in the network, assuming an external population of Poisson excitatory neurons:

$$\boldsymbol{\mu}_a = \begin{bmatrix} K_E g_{aE}\nu_E + K_X g_{aX}\nu_X \\ K_I g_{aI}\nu_I \end{bmatrix} \tag{28}$$

$$\boldsymbol{q}_a^2 = \begin{bmatrix} K_E g_{aE}^2 [\nu_{iE}^2]_i + K_X g_{aX}^2 [\nu_{iX}^2]_i \\ K_I g_{aI}^2 [\nu_{iI}^2]_i \end{bmatrix} \tag{29}$$

Here, we assumed that connectivity is sparse and $[\nu_{ib}^2]_i$ is the average of the squared rates of all neurons in population b [19]. To simplify notations, the voltage heterogeneity of the recurrent excitatory population is considered and the index a is omitted. Inserting Eq.(28)-Eq.(29) to Eq.(27), and with $\mu_i^{ab} = \mu_{ab} + q_{ab} z_i^b$, we get (after some algebra):

$$V_{pas,i} = V_l + \frac{I_{syn,i}}{g_l + G_{syn,i}} = V_l + \frac{I + Q_I z_i}{g_l + G + Q_G z_i} \tag{30}$$

with $z_i \sim \mathcal{N}(0,1)$. The mean and variance of the total time-average currents are

$$\begin{aligned} I &= Kg\nu\{((1+\eta_X)\Delta_E + \gamma\eta\beta\Delta_I) \\ Q_I &= \sqrt{K}g\nu\sqrt{\frac{1}{\nu^2}(([\nu_{iE}^2]_i + [\nu_{iX}^2]_i)\Delta_E^2 + [\nu_{iI}^2]_i\gamma\beta^2\Delta_I^2)} \end{aligned} \tag{31}$$

and of the total time-average conductance:

$$\begin{aligned} G &= Kg\nu((1+\eta_X) + \gamma\eta_I\beta) \\ Q_G &= \sqrt{K}g\nu\sqrt{\frac{1}{\nu^2}(([\nu_{iE}^2]_i + [\nu_{iX}^2]_i) + [\nu_{iI}^2]_i\gamma\beta^2)} \end{aligned} \tag{32}$$

where we defined $\Delta_a = E_a - V_l$ and $K_E = K_X = K, K_I = \gamma K$, $g_I = \beta g_E = \beta g_X = g$. We also defined $\nu_E = \nu, \nu_I = \eta_I\nu, \nu_X = \eta_X\nu$ (for example in cortex often $\eta_I \approx 2$), but one should be careful and remember that both ν, η_X and η_I are all functions of the network parameters.

Small voltage heterogeneity in one compartment models: the balanced regime The issue with one compartment neurons is highlighted by the scaling of the total somatic conductance and currents in Eqs.(31)-(32) with the total synaptic inputs, $Kg\nu$. When there is a balance of the currents, i.e., $((1+\eta_X)\Delta_E + \gamma\eta_I\beta\Delta_I) \approx \frac{1}{\sqrt{K}} \approx 0$ (which is possible as $\Delta_I < 0$), the currents are of order $\sqrt{K}g\nu$, while the total conductance is of order $Kg\nu$. Thus, in the large K limit we get:

$$V_{pas,i} - [V_{pas,i}]_i \approx z_i Q_{tot}/\sqrt{K}$$

and there are very limited sub-threshold voltage heterogeneity. By using the balance condition with Eqs.(31)-(32), it is possible to estimate the SDs of the voltage ($Q_{tot}/\sqrt{K} \approx \frac{Q_I}{G}$):

$$\frac{Q_{tot}}{\sqrt{K}} = \frac{\Delta_E}{\sqrt{K}} \frac{\sqrt{\frac{1}{\nu^2}([\nu_{iE}^2]_i + [\nu_{iX}^2]_i + \frac{[\nu_{iI}^2]_i(1+\eta_x^2)}{\gamma\eta_I^2})}}{(1+\eta_X)(1+|\frac{\Delta_E}{\Delta_I}|)} \tag{33}$$

We can try and estimate the prefactor before the $1/\sqrt{K}$ term in the above equation by using numbers from the biology. For example, if we take $\Delta_E = 55mV, |\frac{\Delta_E}{\Delta_I}| = 2.2, \eta_X = 1, \eta_I = 2, [\nu_{iE}^2]_i = [\nu_{iX}^2]_i = 2\nu^2, [\nu_{iI}^2]_i = 2\eta_I^2\nu^2, \gamma = 1$ we get that $Q_{tot} \approx 55\frac{5\sqrt{2}}{16} \approx 24mV$. Thus, with the $\sqrt{K}$ term in the denominator, the SD of the voltage is around $1mV$ in networks with $K = 400$, and goes to zero as K increases.

Alternatively, if there is no balance of the currents (e.g. see [41]), both the average currents and conductance are large, but on the same order, which is again $\mathcal{O}(\sqrt{K})$ larger than their SDs due to the central limit theorem. Thus, in the large K limit we get that voltage heterogeneity are again $\mathcal{O}(1/\sqrt{K})$ and, and voltage heterogeneity is very limited across neurons.

Finite voltage heterogeneity in extended-like point neurons The problem with one compartment neurons is that the average current and conductance both scale linearly with the total number of pre-synaptic inputs, $Kg\nu$. Despite the balance of the currents, where the mean and heterogeneity of the currents remain on the same order, the total conductance is still an order $\sqrt{K}$ larger than the currents, resulting in limited voltage heterogeneity. One solution to this problem is to scale the synaptic conductance differently for the total conductance and the total currents.

Specifically, in standard balanced networks the synaptic conductance, g, inversely scales with the square root of the of pre-synaptic connections $g = \mathcal{O}(1/\sqrt{K})$ [11] to keep the total currents and their fluctuations on the same order. Instead, we replace g by $g\alpha$ in Eq.(31), but use $g\Gamma$ in Eq.(32). In fact, this is exactly what we get in the phenomenological extended-like model. As we will show below, this approach and the fact that $\Gamma \approx \alpha^2$ (Fig.3C) allows both voltage heterogeneity and total conductance to stay finite in the large K limit.

Our first step is to scale Γ and α in a way that the somatic conductance and currents are independent on the number of connections:

$$\begin{aligned} g\sqrt{K}\nu\alpha &= \mathcal{O}(1) \\ gK\nu\Gamma &= \mathcal{O}(1) \end{aligned} \tag{34}$$

which leads to the scaling of $\Gamma = \alpha^2 = \mathcal{O}(1/K)$. By setting $\alpha = \bar{\alpha}/\sqrt{K}$, where $\bar{\alpha} = \mathcal{O}(1)$, and balancing the excitatory and inhibitory currents, we obtain that the currents across neurons are heterogeneous, of order one, and so is the total conductance. As a result, the mean voltage is:

$$V_{pas,i} - [V_{pas,i}]_i \approx Q_{tot} z_i \tag{35}$$

with

$$Q_{tot} = \frac{\Delta_E \bar{\alpha} g\nu \sqrt{\frac{1}{\nu^2}([\nu_{iE}^2]_i + [\nu_{iX}^2]_i + \frac{[\nu_{iI}^2]_i(1+\eta_x^2)}{\gamma\eta_I^2})}}{g_l + \bar{\alpha}^2 g\nu(1+\eta_X)(1+|\frac{\Delta_E}{\Delta_I}|)}$$

We thus see that in a model in which a pre-synaptic spike affect differently the somatic conductance and the somatic current, and that $\Gamma = \alpha^2$, the mean and SD of the currents and voltages, as well as the somatic conductance, are all finite. They are independent of K. In addition, such a model has finite voltage fluctuations (in time). This eliminates the need for fine-tuning, and similarly to current-based networks, the behavior of the model is well-defined in the limit of large K. However, it should be noted that although $\alpha \to 0$ in the large K limit, this model is not a current-based model, as changes in synaptic inputs affect the neuronal conductance. Finally, while the estimated value of the parameter α from the

multi-compartment models is larger than expected ($\alpha \approx 0.3$, while it should be $\alpha \approx 1/\sqrt{K}$), it can still be beneficial in increasing voltage heterogeneity in finite networks (Fig.S5C).

Statistics of voltage fluctuations Here we calculate the distribution of the voltage SD. By inserting Eqs (24) into (13), one can use a Fokker-Planck formalism to analytically describe the firing rate and the membrane potential distribution of a conductance-based neuron. We follow here [40], and add the neuronal index that arise due to the heterogeneity in synaptic inputs. It is useful to first introduce several quantities, where to simplify notations we write them without their postsynaptic population index:

$$\tau_{pas,i} = \frac{\tau}{1 + \tau(\mu_{aE}(z_i^E) + \mu_{aI}(z_i^I))} \tag{36}$$

$$V_{pas,i} = \tau_{pas,i}(V_l/\tau + \mu_{aE}(z_i^E)E_E + \mu_{aI}(z_i^I)E_I)) \tag{37}$$

that only weakly depend on the disorder in the network because the disorder is always an order $\sqrt{K}$ smaller than the mean μ_{ab}. In fact, in the large K limit Eq.(36) goes to zero and Eq.(37) is independent of K.

Furthermore, we also introduce two voltage quantities:

$$E_s = \frac{\sigma_{aE}^2 E_E + \sigma_{aI}^2 E_I}{\chi}$$

$$E_d = \sigma_{aE}\sigma_{aI}(E_E - E_I)\chi$$

and

$$\chi = \frac{1}{\sigma_{aE}^2 + \sigma_{aI}^2}$$

which none of them depends on the quenched disorder. Finally, we also introduce the dimensionless parameter $\gamma_i = \frac{2\chi}{\tau_{pas,i}}$, which needs to be large in order for the Gaussianity assumption to hold.

Using a linear change of variables from voltage to a dimensionless variable, x_i:

$$x_i = (V_i - V_{pas,i})\sqrt{\frac{\gamma_i}{(V_{pas,i}^2 - E_s)^2 + E_d^2}} \tag{38}$$

with x_{th} and x_r corresponding to placing V_{th} and V_r in Eq.(38). It is then possible to write the equilibrium distribution of the FP equation for $f(x_i)$:

$$-\nu\tau_{pas,i}\Theta(x_i - x_r) = \frac{d}{dx}(a_i^2 x_i^2 - 2a_i b_i x_i + 1)f(x_i) + x_i f(x_i) \tag{39}$$

with $a_i = \frac{1}{\sqrt{\gamma_i}}$ and $b_i = \frac{E_s - V_{pas,i}}{\sqrt{(E_S - V_{pas,i})^2 + E_D^2}}$. Integrating Eq.(39) after multiplying it with x_i^n gives, after notorious algebra, the moments of the distribution. The first and second moments of the voltage yields:

$$\bar{V}_i = V_{pas,i}(s_a) - \tau_{pas,i}(s_a)\nu_i(s_a) \tag{40}$$

and

$$\sigma_{V,i}^2 = \sigma_{pas,i}^2(s_a) - \tau_{pas,i}\nu_i V_-(\bar{V}_i(s_a) + \frac{V_+}{2}(\frac{\gamma_i}{1-\gamma_i}) + \frac{E_s - V_{pas,i}}{\gamma_i}) \tag{41}$$

with the variance of a passive conductance-based IF neuron given by $\sigma_{pas,i}^2 = \frac{(E_s - V_{pas.i})^2 + E_D^2}{\gamma_i - 1}$. One should note that as γ_i scales with the synaptic strength $1/g_{ab}$, we get that when g_{ab} is small, and for which the Gaussianity assumption is valid, the leading order of the fluctuations takes the simple form

$$\sigma_{V,i}^2 = \sigma_{pas,i}^2(s_a) - \tau_{pas,i}\nu_i V_-(\bar{V}_i(s_a) - \frac{V_+}{2}) \tag{42}$$

Thus, both the mean and the SD of the voltage for conductance-based IF neurons (Eq.(40),(42)) have the same form as the mean and SD of the current-based IF neurons. It is the mean or SD of the passive neuron, together with another term that is proportional to the rate of the neuron.

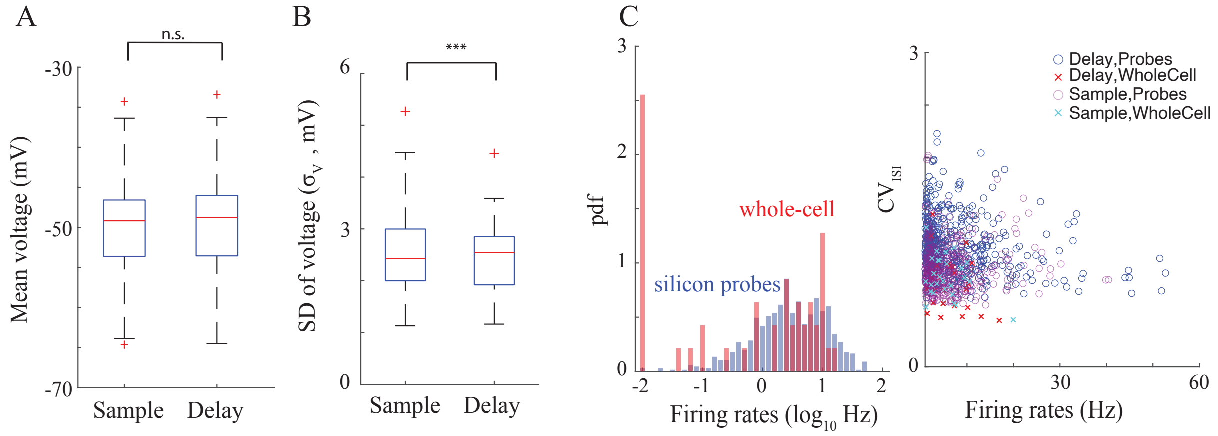

Figure S 1: Statistics of supra and sub-threshold activity of ALM neurons during different behavioral periods. A. Mean voltage of the 47 ALM neurons during sample and delay epochs. **B.** SD of voltage. **C.** Left: Firing rate distributions of putative excitatory neurons recorded using silicon probes (blue, same distribution as in Fig.1C) and whole-cell recordings (red). Right: CV_{ISI} for sample and delay periods of excitatory neurons based on whole-cell or silicon probes recordings.

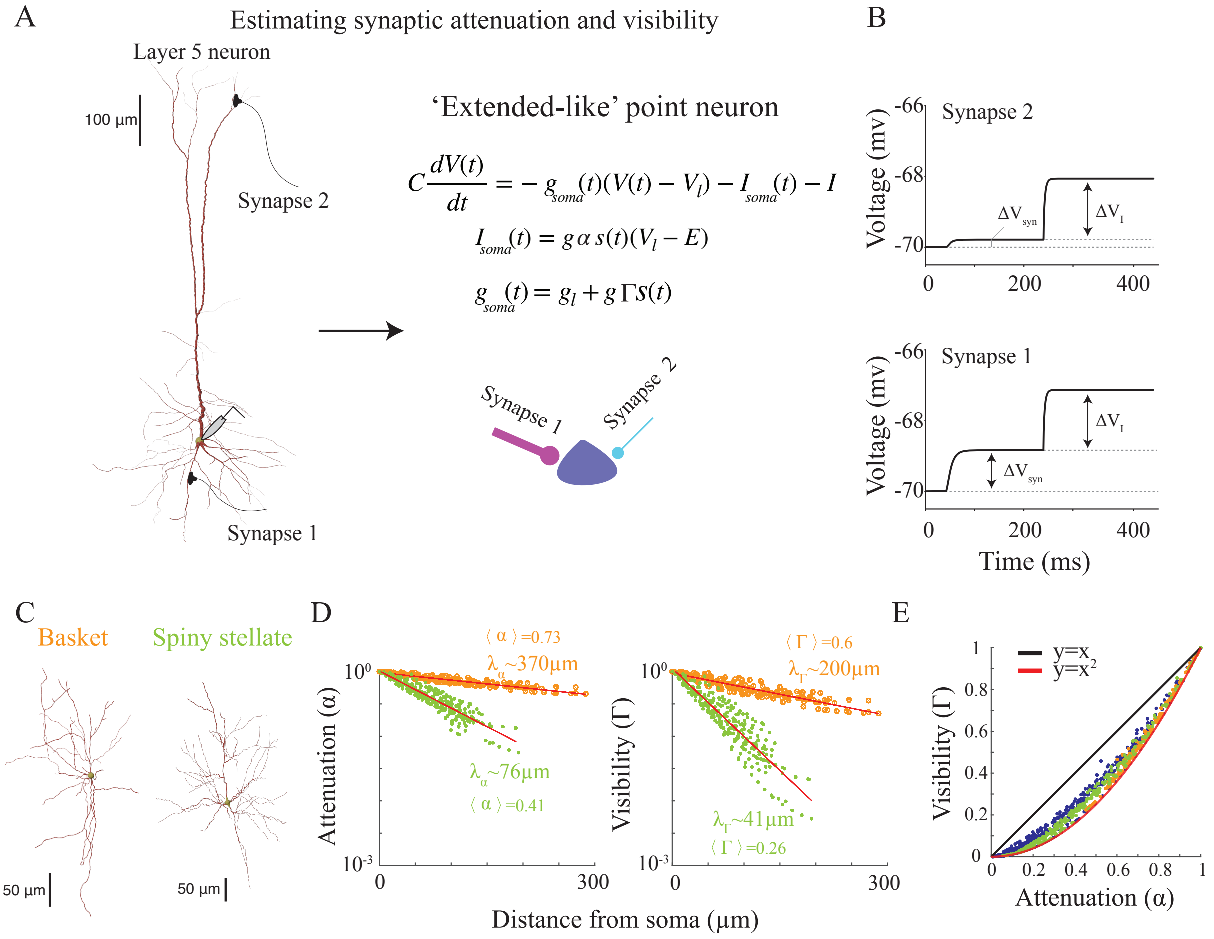

Figure S 2: Estimation of the attenuation and visibility for synapses in a multi-compartment model. **A.** Estimation of the attenuation and visibility parameters of the phenomenological point neuron model from a simulation of a multi-compartment model. Left: reconstruction of the simulated layer 5 pyramidal neuron. **B.** The voltage dynamics of the 'extended-like' point neuron. Right: The voltage traces of the two example synapses. First, we activate a synapse in a multi-compartment model and measure the change in voltage at the soma (ΔV_{syn}). We then inject a step current at the soma (ΔI) and measure the voltage change (ΔV_I). The comparison of ΔV_{syn} and ΔV_I in the multi-compartment model and the point neuron is used to estimate the visibility (Γ) and attenuation (α) parameters; see Methods. We repeat this process for every synapse along the dendritic tree. **C.** Same as in (A), but for a multi-compartment model of basket (orange) and spiny stellate (green) cells. **D.** Left: Attenuation vs. the distance of the synapse from soma for the cell in (C). Red line: fit to an exponential decay. Right: Same as left, but for the visibility parameter. Note that the decay rate of the visibility is around twice the decay rate of the attenuation parameter. **E.** The estimated visibility against attenuation for synapses in the three cell types. Note that the visibility always decays faster than the attenuation, and that for an infinite cylinder $\Gamma \approx \alpha^2$ (Fig.S3C; [43]).

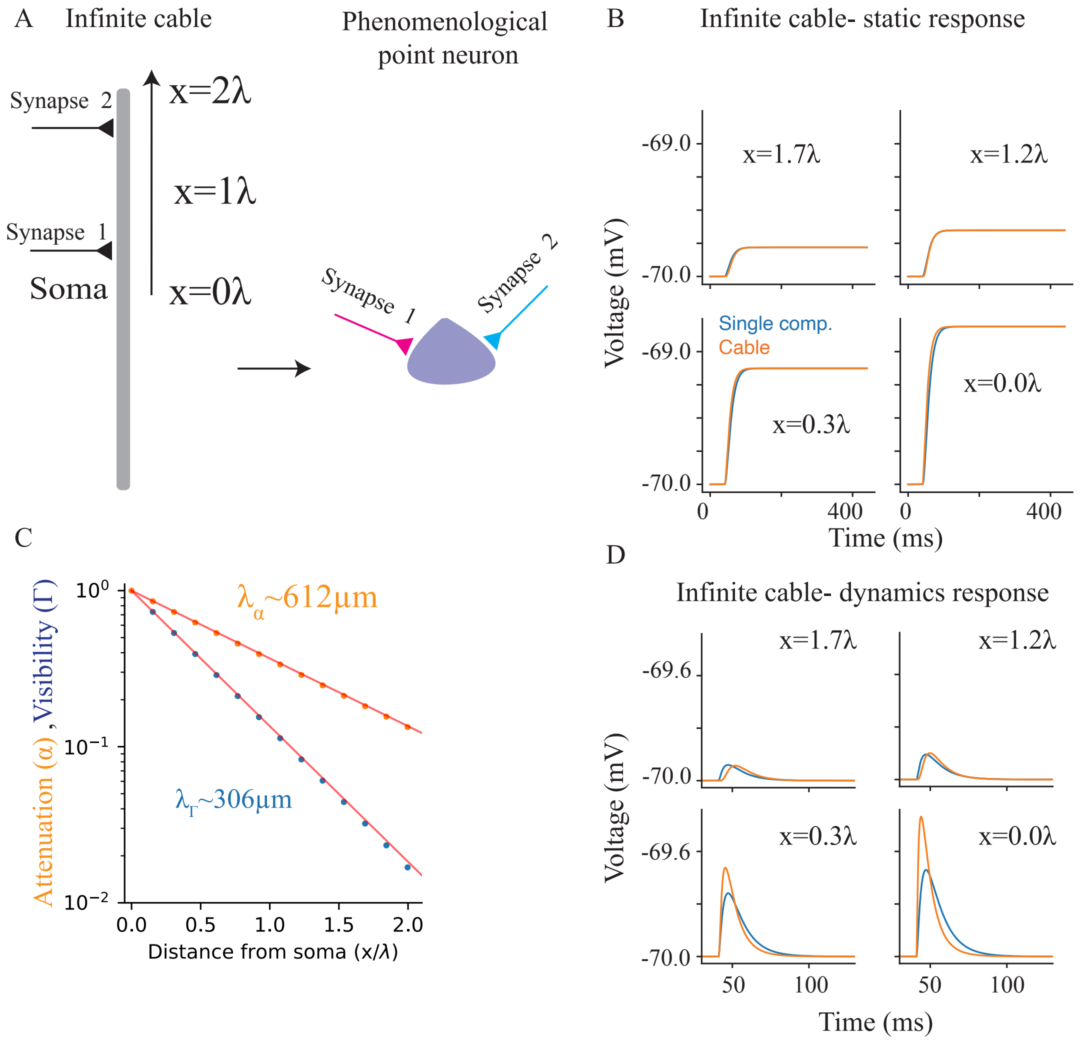

Figure S 3: Mapping synaptic activity in an infinite cable to activity of an extended-like point neuron. A. Left: a cartoon of the simulated infinite cylinder (with length and diameter of 9500 μm and 1.5μm respectively, and specific membrane resistance of 10,000 Ωcm^2) with a synapse that is located along the cylinder. Right: the extended-like point neuron. **B.** Four examples of the static response of the cable (orange) to activating a synapse at different locations along the cable, and the corresponding mapped synapse when activated on the point neuron (blue). **C.** The attenuation (α) and visibility (Γ) of the synapse against the location of the synapse. Note that in an infinite cylinder $\alpha \propto e^{-x/\lambda}$ and $\Gamma \propto e^{-x/2\lambda}$ and thus $\lambda_\alpha = 2\lambda_\Gamma$ [43]). **D.** Four examples of the dynamic response of the cable (orange) to activating a synapse (alpha function) at different locations along the cable, and the corresponding mapped synapse of the point neuron (blue). Note that the differences in the voltage are a result of estimating α, Γ using the static response of the neurons.

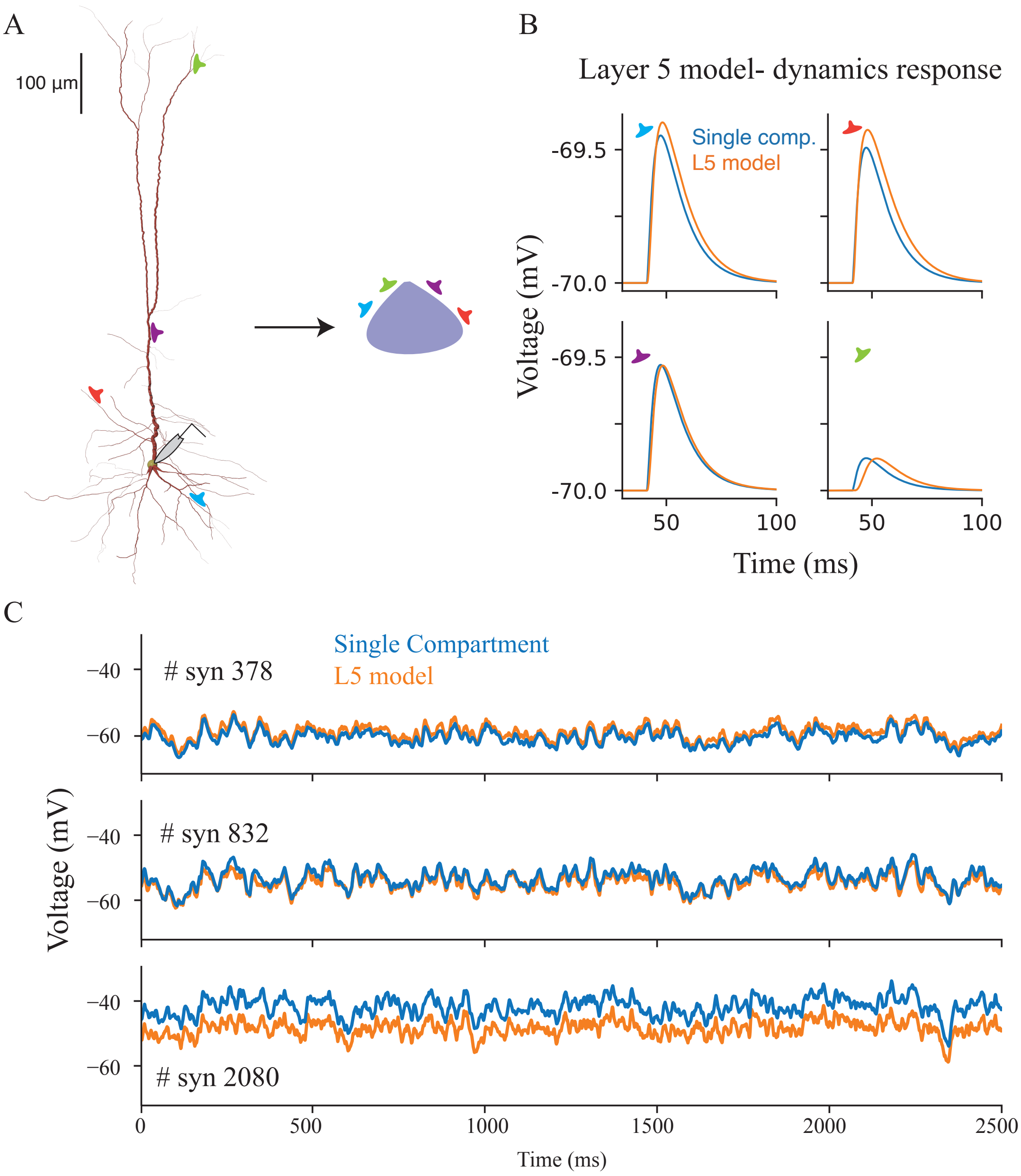

Figure S 4: Mapping synaptic activity in layer 5 neuron to activity of an extended-like point neuron. A. Mapping of the layer 5 neuron (as in main text). **B.** Four examples of the dynamic response of the layer 5 neuron (orange) to activating a synapse (double-exponent) at different locations along the cable, and the corresponding mapped synapse (α, Γ) of the point neuron (blue). Note that the differences in the voltage are a result of estimating α, Γ using the static response of the neurons. **C.** Voltage responses for random activation of excitatory and inhibitory synapses (as in Fig.3C, case shown are of 378, 832 and 2080 synapses) in the full model and single compartment where all synapses are mapped according to their α, Γ in the full model.

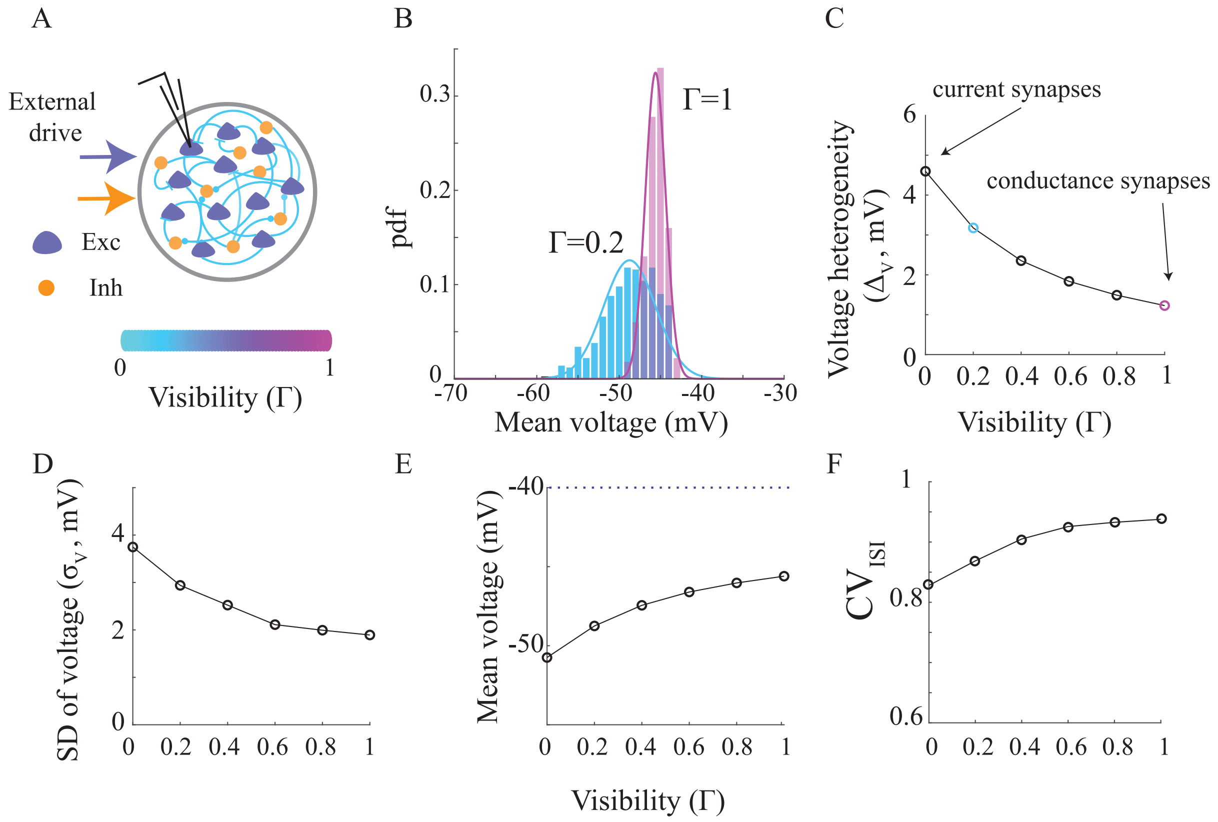

Figure S 5: The effect of the visibility of the conductance on sub-threshold heterogeneity and spiking irregularity in a recurrent network. A. A cartoon of a network of integrate-and-fire neurons with a mixture of current and conductance synapses. The case in which $\Gamma = 1$ corresponds to a pure conductance-based synapse, while the case of $\Gamma = 0$ is what is known in the literature as current-based synapses, in which synaptic changes do not affect the conductance of the neuron (see Eq.(12) and Methods). For these simulations, we assumed the same values of visibility and attenuation for all excitatory, inhibitory and external synapses and we merged the attenuation parameter together with the synaptic strength (g in Eq. (12)). We varied the level of Γ while maintaining the average firing rates for excitatory and inhibitory populations. **B.** Distribution of time-average voltage of excitatory neurons in a network with $\Gamma = 0.2$ (cyan) and of pure conductance-based synapses, $\Gamma = 1$ (magenta). Threshold at $-40mV$ **C.** Voltage heterogeneity vs. the visibility parameter. Colored circles are the SDs of the distributions of the examples in (B). **D-F.** SD of (single neuron) voltage fluctuations (D), mean voltage (E) and coefficient of variation of the inter-spike-intervals (CV_{ISI}) (F) against the visibility parameter. Note that the SD of the voltage and the CV_{ISI} varied with Γ in opposite directions. In all panels the mean rate of the excitatory and inhibitory neurons was kept constant ($4Hz$ and $9Hz$, respectively) when changing the visibility parameter by adapting the external inputs.

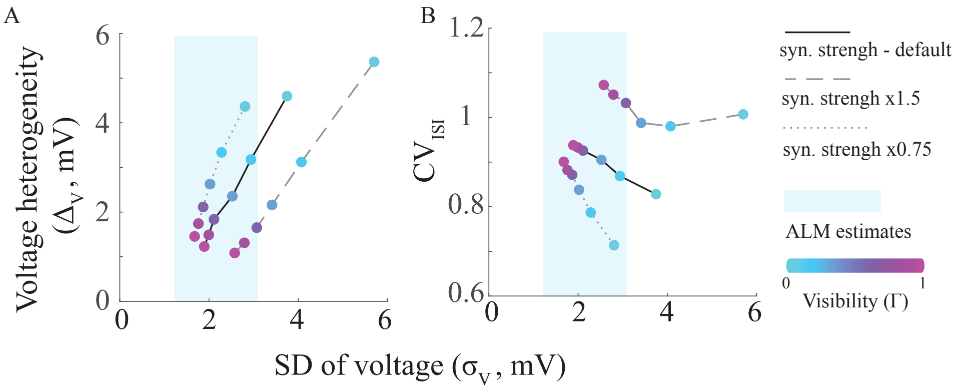

Figure S 6: Visibility and network parameters in networks of extended-like point neurons that are consistent with ALM data. Sweep of visibility and network parameters to find visibility parameters that are consistent with ALM data. Solid lines: same figures as in Fig.S5C,D,F, but plotted against the SD of the voltage. The value of each circle is obtained from a network simulation with a different visibility parameter (ranging from zero to one, with jumps of 0.2). Dashed lines: same as solid lines but for a different set of network parameters. To keep the average firing rate in the network constant, and consistent with ALM data, we increased the synaptic strengths by a factor of 1.5 ($1.5\bar{g}_{ab}$ and $1.5\bar{I}_{ext,a}$ of the parameters in Supplementary Table 1). As a result, voltage fluctuations in the network increased, while the mean rate was not changed. Dotted lines: Same as dashed lines but with a 0.75 factor. Cyan: mean±SD of the estimated voltage SD in the population of ALM neurons (see Fig.7E, right). Large voltage heterogeneity is limited by the level of voltage fluctuations and the CV_{ISI} of neurons in the network. Large voltage heterogeneity together with high CV_{ISI} in the range of the SD of the voltage of ALM neurons can be achieved for ranges of $\Gamma = 0.2 - 0.4$. Note that larger visibility parameters for $\sigma_V \approx 2mV$ would decrease both the voltage heterogeneity and the CV_{ISI} to values that are inconsistent with ALM data. **A.** Voltage heterogeneity against voltage SD. **B.** CV_{ISI} against voltage SD.

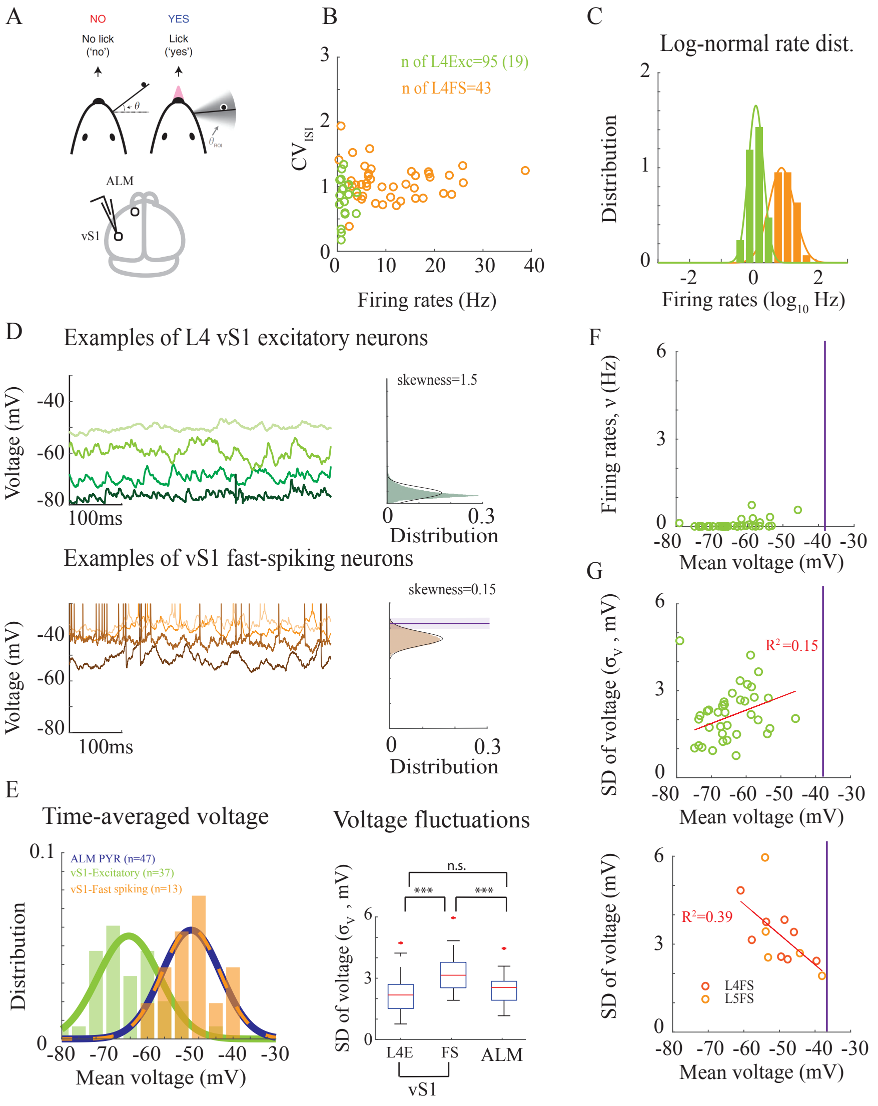

Figure S 7: Supra- and sub-threshold statistics of vS1 neurons in mice performing a Go/NoGo task. **A.** Top: behavioral task. The mouse had to detect the pole location during the sampling period and reported its location by either licking or not. Bottom: recording area. See [38] for details. **B.** CV_{ISI} against neuronal firing rates for neurons in layer 4 (L4). Most of the L4 excitatory neurons were quiescent (only 19/95 neurons that spiked are presented). **C.** Probability density function (pdf) of the log-rates. Solid lines: fit to a Gaussian distribution. **D.** Top: Examples of layer 4 excitatory neurons. Bottom: Fast-spiking neurons. Left: Activity of example neurons during the first half-second of a whisking episode. Right: Sub-threshold voltage distribution of one of the neurons. Solid line: fit to a Gaussian distribution. **E.** Left: Probability density function (pdf) of time-average voltage for all recorded neurons. Solid lines: fit to a Gaussian distribution. Right: SD of (single neuron) voltage fluctuations for all recorded neurons. **F.** Firing rates vs. mean voltage for the excitatory neurons. **G.** SD of (single neuron) voltage fluctuations against the mean voltage. Top: layer 4 excitatory neurons. Bottom: Fast-spiking neurons in layer 4 and Layer 5. Red line: linear regression. In (B-C) Extracellular and intracellular recordings. In (D-G) whole-cell recordings. In (B-C,E-G) analysis during non-whisking periods (see also Fig.S9). Panel A adapted from O'Connor, D., Hires, S., Guo, Z. et al. Neural coding during active somatosensation revealed using illusory touch. Nat Neurosci 16, 958–965 (2013). https://doi.org/10.1038/nn.3419.

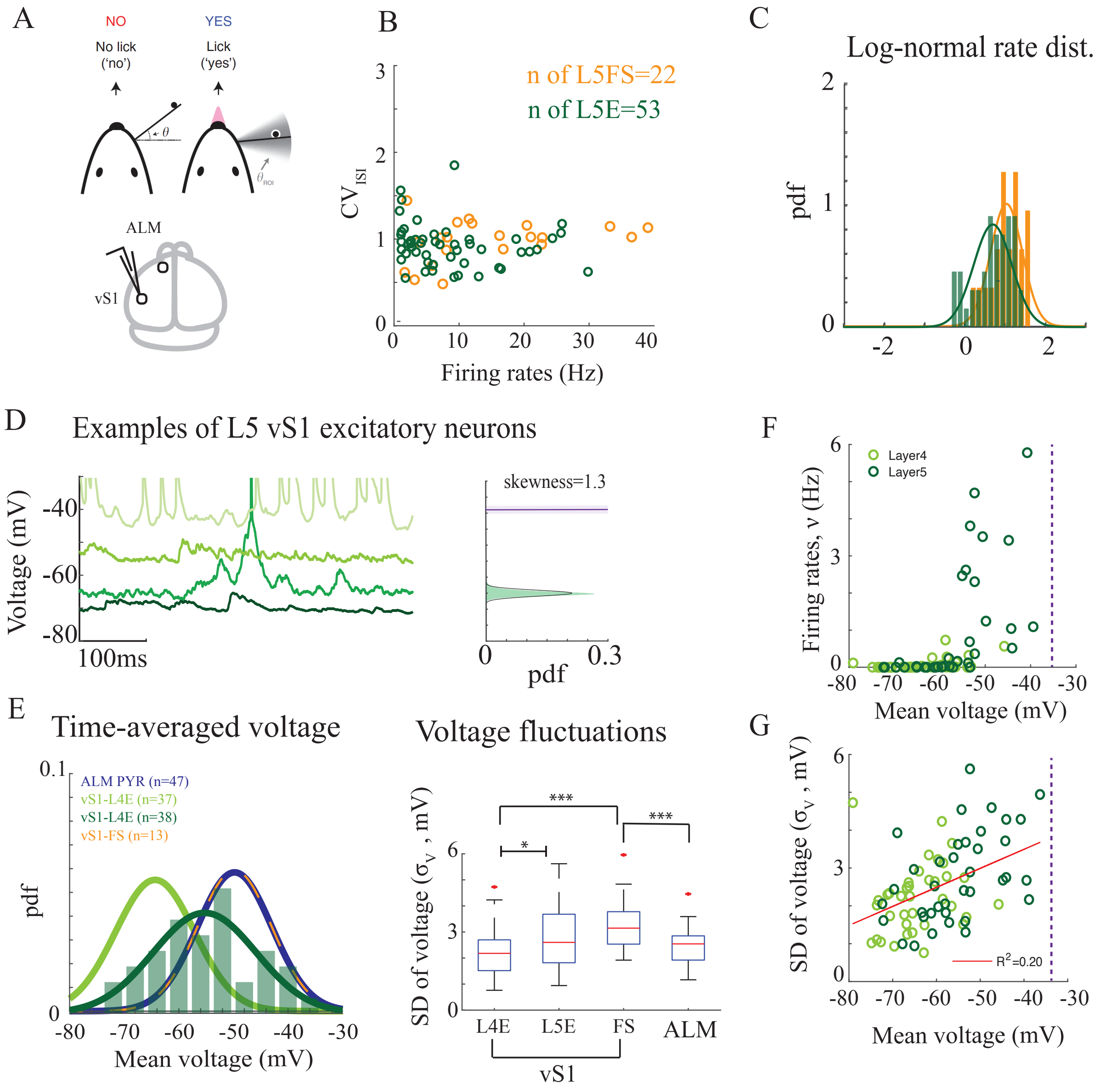

Figure S 8: Supra and sub-threshold statistics of layer 5 S1 neurons in mice performing a Go/NoGo task. A. Top: behavioral task. Bottom: recording area. **B.** CV_{ISI} against neuronal firing rates for neurons in layer 5. **C.** Probability density function (pdf) of the log-rates. Solid lines: fit to a Gaussian distribution. **D.** Example of layer 5 excitatory neurons. Left: Activity of example neurons during the first 0.5 second of a whisking episode. Right: Sub-threshold voltage distribution of one of the neurons. Solid line: fit to a Gaussian distribution. **E.** Left: Probability density function (pdf) of time-average voltage for all recorded neurons, including layer 5 excitatory neurons in vS1. Solid lines: fit to a Gaussian distribution. Right: SD of (single neuron) voltage fluctuations for all recorded neurons. **F.** Firing rates vs. mean voltage for the excitatory neurons. **G.** SD of (single neuron) voltage fluctuations against the mean voltage. Red line: linear regression. In (B-C) Extracellular and intracellular recordings. In (D-G) whole-cell recordings. In (B-C,E-F) analysis during non-whisking periods. Panel A adapted from O'Connor, D., Hires, S., Guo, Z. et al. Neural coding during active somatosensation revealed using illusory touch. Nat Neurosci 16, 958–965 (2013). https://doi.org/10.1038/nn.3419.

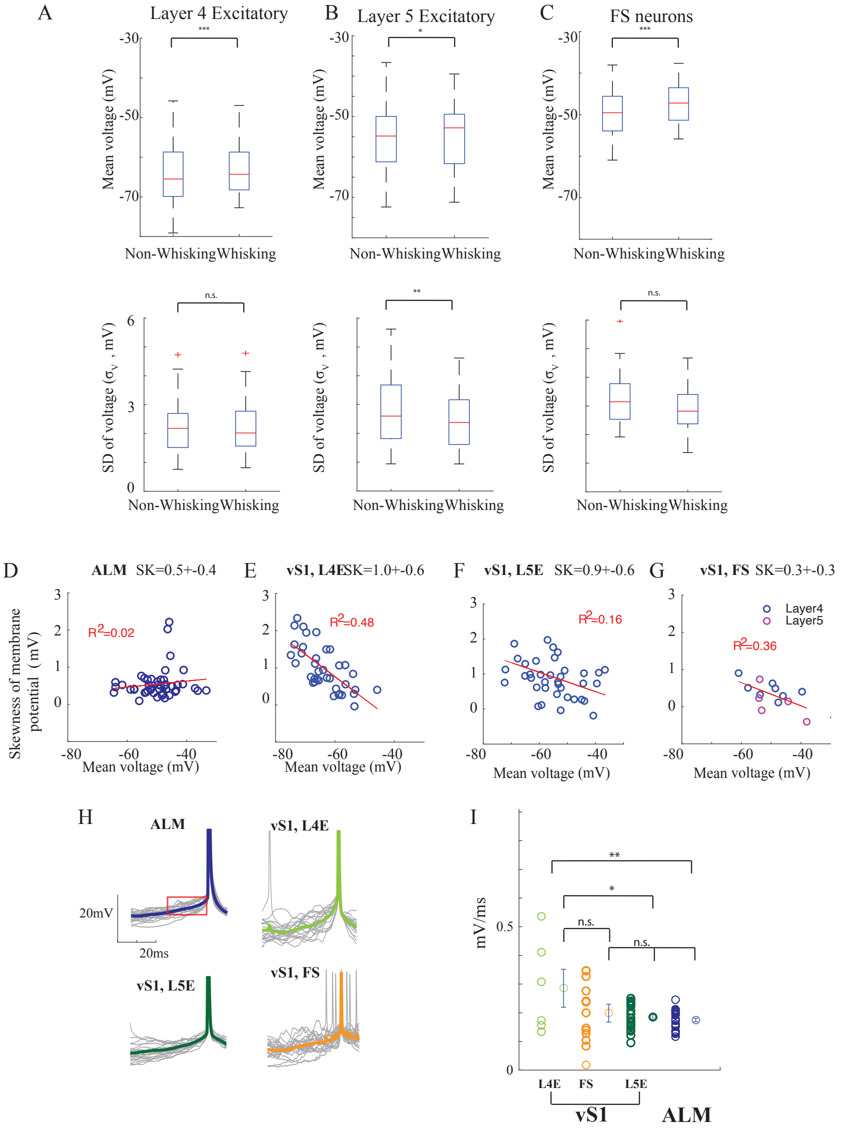

Figure S 9: Mean, SD and skewness of the sub-threshold voltage distributions and membrane potential trajectory for evoking action potentials. **A.** Top: mean voltage for non-whisking and whisking periods during the sample period for layer 4 excitatory neurons (n=37) in vS1. Bottom: SD of voltage of the same neurons. **B.** Same as (A) but for layer 5 neurons (n=38). **C.** Same as (A) but for FS neurons (n=13). Statistical tests in this figure are based on paired two-sided Wilcoxon signed rank test **D-G.** Skewness of the voltage of a neuron, $V_i(t)$, defined as $SK_i = \frac{\frac{1}{T}\sum_t (V_i(t)-\bar{V_i})^3}{\left(\sqrt{\frac{1}{T}\sum_t (V_i(t)-\bar{V_i})^2}\right)^3}$, against the mean voltage for ALM (D) and vS1 neurons (F-G). Population-average skewness, $SK = \frac{1}{N}\sum SK_i$, of each population is given in the title. **H.** Thirty superimposed spikes for example neurons. Spike-triggered average membrane potential dynamics (excluding spikes) were calculated over the first 30 trials for each spiking neuron. **I.** The increase in membrane potential in the 20 ms before the spike (red rectangle in (E), given in units of mV/ms) is significantly steeper for layer 4 excitatory neurons than for layer 5 excitatory neurons in vS1 and in ALM. Each circle is a neuron and the mean$\pm$*sem* is plotted for each population. Neurons in vS1 with at least 30 spikes during non-whisking periods were analyzed. vS1 layer 4 excitatory: n=6; vS1 layer 5 excitatory n=20; vS1 fast spiking: n=13; ALM excitatory neurons: n=24. Statistical test: unpaired Student t-test (see also [69].)

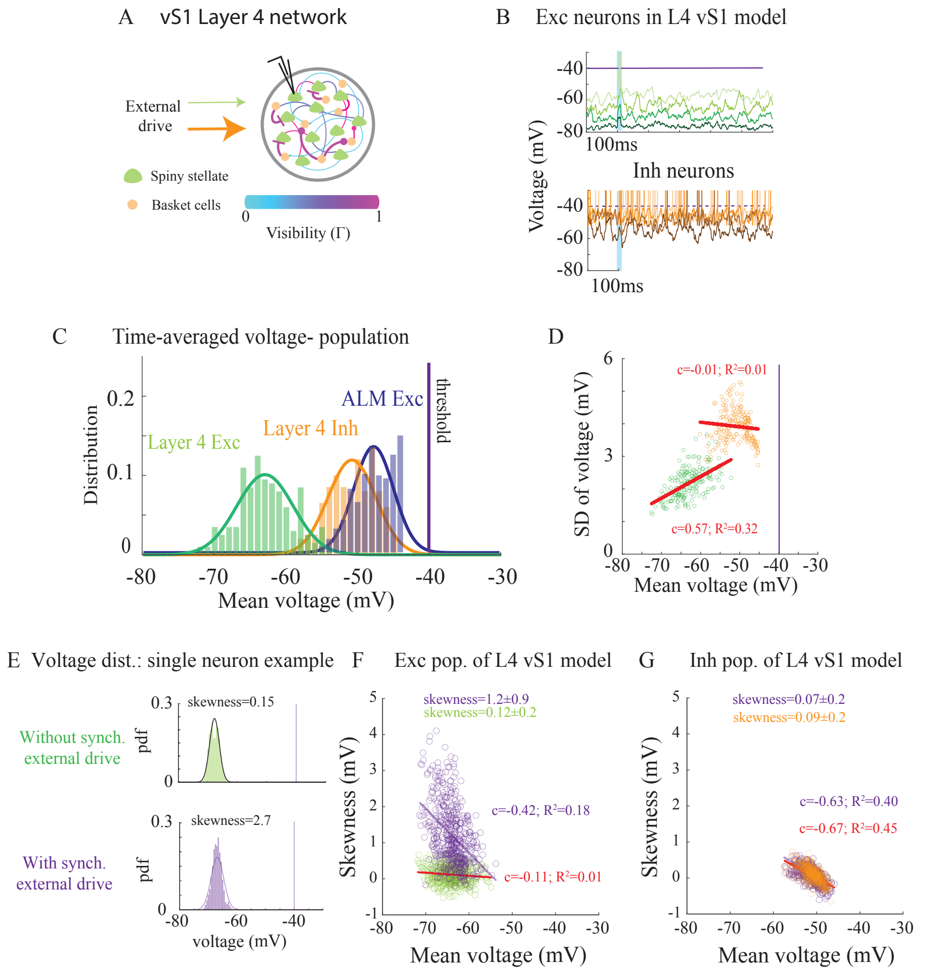

Figure S 10: Model of layer 4 vS1 network. **A.** Diagram of an vS1 layer 4 network model with attenuation and visibility parameters estimated from multi-compartment models of spiny stellate (green) and basket (orange) cells. The main difference with the ALM network in Fig.4 is in the weak external drive to the excitatory population, which lead to an unbalanced excitatory population. **B.** Excitatory neurons in the model fired as a result of short $10ms$ synchronous external drive to all neurons (cyan). Note that the excitatory neurons in the model fire only as a result of the synchronous drive. **C.** Probability density function (pdf) of time-average voltage for the neurons in the vS1 and ALM networks. Compare with Compare with Fig.S7E. **D.** Voltage SD against mean voltage across the population for excitatory (green) and inhibitory (orange) neurons. Red: linear fit with a slope (c; mean-SD correlation) and goodness-of-fit (R^2). Compare with Fig.S7G. **E.** Example of an excitatory neuron in layer 4 vS1 network model without (top-green) and with (bottom- purple) synchronous external drive (see Methods). Without external drive the voltage distribution is well-fitted with a Gaussian distribution (solid line; low skewness). **F.** Skewness of excitatory neurons without (green) and with (purple) synchronous external drive. Red line: linear fit in a network without synchronous external drive. Correlations between skewness and mean voltage of neurons in the network are low (small c). Purple line: linear fit in a network with synchronous external drive. Skewness and mean voltage of neurons in the model is negatively correlated (negative c). Note that the synchronous external drive mainly affects the hyperpolarized excitatory neurons. Compare with Fig.S7E. **G.** Same as (F), but for the inhibitory population, which are more depolarized than the excitatory neurons. The external drive only weakly affects the skewness of the neurons.

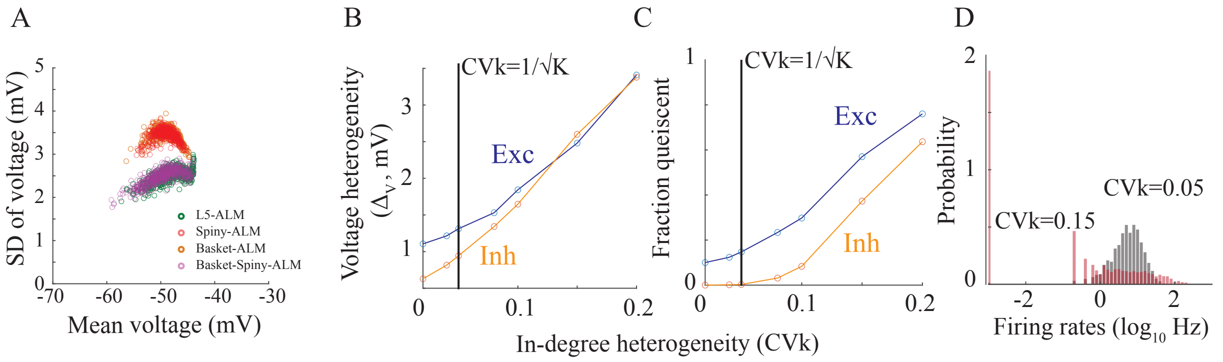

Figure S 11: Simulations of attenuation and visibility parameters estimated for vS1 with ALM network and the effect of heterogeneous in-degree connectivity on membrane potential heterogeneity. A. Voltage statistics of neurons in a network of extended-like neurons is similar when the visibility and attenuation parameters are estimated from a layer 5 pyramidal neuron or from a spiny stellate neuron. SD of voltage fluctuations against the mean voltage of 500 neurons in a network with layer 5-basket cells (green and orange) and spiny stellate-basket cells (purple and red). Same network parameters as in Fig.4A-E. **B-D.** Increase of in-degree voltage heterogeneity increases the voltage heterogeneity (**B**), but pushes the network out of the excitation-inhibition balanced regime, as shown by the increase in quiescent neurons (**C**) and the deviation from the log-normal firing rates (**D**). Same parameters as in Fig.2, with $K = K_E = K_I = 400$. Coefficient of variation of the number of pre-synaptic inputs per neuron, CV_K, see [57]. The fraction of quiescent neurons is small below $CV_K \approx 1/\sqrt{K}$, corresponding to $CV_K = 0.05$, with $K = 400$ (vertical black line in (B-C).

	Neuron parameters	Fig.S 5-6	Fig.S11
dt	simulation time step	0.5 ms	0.1 ms
g_l	neuronal conductance	$0.1 mS/cm^2$	
C	neuronal capacitance	$1 \mu F/cm^2$	
V_{th}	spike threshold	-40mV	
V_r	voltage reset after spike	-52mV	
V_l	voltage reset after spike	-55mV	
E_E	reversal potential of exc synapses	0mV	
E_I	reversal potential of inh synapses	-80mV	
	**Network parameters**		
τ_{syn}	synaptic time constant	3 ms	
N	number of neurons	10000	1800
N_E	number of excitatory neurons	5000	1600
N_I	number of inhibitory neurons	5000	200
K_E	average number of exc synapses to a neuron	400	50
K_I	average number of inh synapses to a neuron	400	50
p_a	connection probability	K_a/N_a	
$\bar{g}_{EE}$	exc to exc synaptic weight	$0.00133 mS/cm^2$	0.0026
$\bar{g}_{IE}$	exc to inh synaptic weight	$0.02 mS/cm^2$	0.039
$\bar{g}_{II}$	inh to inh synaptic weight	$0.133 mS/cm^2$	0.18
$\bar{g}_{EI}$	inh to exc synaptic weight	$0.08 mS/cm^2$	0.156
α_{ij}^{ab}	attenuation parameter	1	estimated
ρ_{ij}^{ab}	mixing parameter	varied	estimated
$\bar{I}_{ext,E}$	external input to exc	varied	$0.001 \mu A/cm^2$
$\bar{I}_{ext,I}$	external input to inh	varied	$0.01 \mu A/cm^2$
$q_{ext,E}$	disorder in external input to exc	$0.008 \mu A/cm^2$	
$q_{ext,I}$	disorder in external input to inh	$0.005 \mu A/cm^2$	
ρ_{ext}	average mixing parameter of ext inputs	varied	0
g_s	selective inh-to-inh synapses	0	0
$\bar{I}_{ext,E}^1$	external input during delay period to exc	0	0
$\bar{I}_{ext,I}^1$	external input during delay period to inh	0	0

Supplementary Table 1: Network simulation parameters for supplementary figures.

References

[1] Softky, W. R. & Koch, C. The highly irregular firing of cortical cells is inconsistent with temporal integration of random epsps. *Journal of neuroscience* **13**, 334–350 (1993).

[2] Shadlen, M. N. & Newsome, W. T. The variable discharge of cortical neurons: implications for connectivity, computation, and information coding. *Journal of neuroscience* **18**, 3870–3896 (1998).

[3] Compte, A. *et al.* Temporally irregular mnemonic persistent activity in prefrontal neurons of monkeys during a delayed response task. *Journal of neurophysiology* **90**, 3441–3454 (2003).

[4] Griffith, J. & Horn, G. An analysis of spontaneous impulse activity of units in the striate cortex of unrestrained cats. *The Journal of Physiology* **186**, 516 (1966).

[5] Hromádka, T., DeWeese, M. R. & Zador, A. M. Sparse representation of sounds in the unanesthetized auditory cortex. *PLoS biology* **6**, e16 (2008).

[6] O'Connor, D. H., Peron, S. P., Huber, D. & Svoboda, K. Neural activity in barrel cortex underlying vibrissa-based object localization in mice. *Neuron* **67**, 1048–1061 (2010).

[7] Buzsáki, G. & Mizuseki, K. The log-dynamic brain: how skewed distributions affect network operations. *Nature Reviews Neuroscience* **15**, 264–278 (2014).

[8] Ahmadian, Y. & Miller, K. D. What is the dynamical regime of cerebral cortex? *Neuron* (2021).

[9] Hires, S. A., Gutnisky, D. A., Yu, J., O'Connor, D. H. & Svoboda, K. Low-noise encoding of active touch by layer 4 in the somatosensory cortex. *Elife* **4**, e06619 (2015).

[10] Stringer, C. *et al.* Spontaneous behaviors drive multidimensional, brainwide activity. *Science* **364** (2019).

[11] van Vreeswijk, C. & Sompolinsky, H. Chaos in neuronal networks with balanced excitatory and inhibitory activity. *Science* **274**, 1724–1726 (1996).

[12] Amit, D. J. & Brunel, N. Model of global spontaneous activity and local structured activity during delay periods in the cerebral cortex. *Cerebral cortex (New York, NY: 1991)* **7**, 237–252 (1997).

[13] Brunel, N. Dynamics of sparsely connected networks of excitatory and inhibitory spiking neurons. *Journal of computational neuroscience* **8**, 183–208 (2000).

[14] Hertz, J., Lerchner, A. & Ahmadi, M. Mean field methods for cortical network dynamics. In *International School on Neural Networks, Initiated by IIASS and EMFCSC*, 71–89 (Springer, 2003).

[15] Vogels, T. P., Rajan, K., Abbott, L. F. *et al.* Neural network dynamics. *Annual review of neuroscience* **28**, 357 (2005).

[16] Destexhe, A., Rudolph, M., Fellous, J.-M. & Sejnowski, T. J. Fluctuating synaptic conductances recreate in vivo-like activity in neocortical neurons. *Neuroscience* **107**, 13–24 (2001).

[17] Van Vreeswijk, C. & Sompolinsky, H. Irregular activity in large networks of neurons. *Methods and models in neurophysics. Amsterdam: Elsevier* (2005).

[18] Renart, A. *et al.* The asynchronous state in cortical circuits. *science* **327**, 587–590 (2010).

[19] Roxin, A., Brunel, N., Hansel, D., Mongillo, G. & van Vreeswijk, C. On the distribution of firing rates in networks of cortical neurons. *The Journal of neuroscience* **31**, 16217–16226 (2011).

[20] Hansel, D. & Mato, G. Short-term plasticity explains irregular persistent activity in working memory tasks. *Journal of Neuroscience* **33**, 133–149 (2013).

[21] Rosenbaum, R., Smith, M. A., Kohn, A., Rubin, J. E. & Doiron, B. The spatial structure of correlated neuronal variability. *Nature Neuroscience* **20**, 107–114 (2017).

[22] Darshan, R., Van Vreeswijk, C. & Hansel, D. Strength of correlations in strongly recurrent neuronal networks. *Physical Review X* **8**, 031072 (2018).

[23] Hansel, D. & van Vreeswijk, C. The mechanism of orientation selectivity in primary visual cortex without a functional map. *Journal of Neuroscience* **32**, 4049–4064 (2012).

[24] Pehlevan, C. & Sompolinsky, H. Selectivity and sparseness in randomly connected balanced networks. *PloS one* **9**, e89992 (2014).

[25] Pattadkal, J. J., Mato, G., van Vreeswijk, C., Priebe, N. J. & Hansel, D. Emergent orientation selectivity from random networks in mouse visual cortex. *Cell reports* **24**, 2042–2050 (2018).

[26] Haider, B., Duque, A., Hasenstaub, A. R. & McCormick, D. A. Neocortical network activity in vivo is generated through a dynamic balance of excitation and inhibition. *Journal of Neuroscience* **26**, 4535–4545 (2006).

[27] Okun, M. & Lampl, I. Instantaneous correlation of excitation and inhibition during ongoing and sensory-evoked activities. *Nature neuroscience* **11**, 535–537 (2008).

[28] Mahrach, A., Chen, G., Li, N., van Vreeswijk, C. & Hansel, D. Mechanisms underlying the response of mouse cortical networks to optogenetic manipulation. *Elife* **9**, e49967 (2020).

[29] Sanzeni, A. *et al.* Inhibition stabilization is a widespread property of cortical networks. *Elife* **9**, e54875 (2020).

[30] Kim, C. M., Finkelstein, A., Chow, C. C., Svoboda, K. & Darshan, R. Distributing task-related neural activity across a cortical network through task-independent connections. *Nature Communications* **14**, 2851 (2023).

[31] Gutnisky, D. A. *et al.* Mechanisms underlying a thalamocortical transformation during active tactile sensation. *PLoS computational biology* **13**, e1005576 (2017).

[32] Tan, A. Y., Chen, Y., Scholl, B., Seidemann, E. & Priebe, N. J. Sensory stimulation shifts visual cortex from synchronous to asynchronous states. *Nature* **509**, 226–229 (2014).

[33] Larkum, M. Are dendrites conceptually useful? *Neuroscience* (2022).

[34] Guo, Z. V. *et al.* Maintenance of persistent activity in a frontal thalamocortical loop. *Nature* **545**, 181–186 (2017).

[35] Inagaki, H. K., Fontolan, L., Romani, S. & Svoboda, K. Discrete attractor dynamics underlies persistent activity in the frontal cortex. *Nature* **566**, 212–217 (2019).

[36] Guo, Z. V. *et al.* Flow of cortical activity underlying a tactile decision in mice. *Neuron* **81**, 179–194 (2014).

[37] Shinomoto, S., Shima, K. & Tanji, J. Differences in spiking patterns among cortical neurons. *Neural computation* **15**, 2823–2842 (2003).

[38] Yu, J., Gutnisky, D. A., Hires, S. A. & Svoboda, K. Layer 4 fast-spiking interneurons filter thalamocortical signals during active somatosensation. *Nature neuroscience* **19**, 1647–1657 (2016).

[39] Destexhe, A., Rudolph, M. & Paré, D. The high-conductance state of neocortical neurons in vivo. *Nature reviews neuroscience* **4**, 739–751 (2003).

[40] Richardson, M. J. Effects of synaptic conductance on the voltage distribution and firing rate of spiking neurons. *Physical Review E* **69**, 051918 (2004).

[41] Sanzeni, A., Histed, M. H. & Brunel, N. Emergence of irregular activity in networks of strongly coupled conductance-based neurons. *Physical Review X* **12**, 011044 (2022).

[42] Rall, W. Theoretical significance of dendritic trees for neuronal input-output relations. *Neural theory and modeling* 73–97 (1964).

[43] Koch, C., Douglas, R. & Wehmeier, U. Visibility of synaptically induced conductance changes: theory and simulations of anatomically characterized cortical pyramidal cells. *Journal of Neuroscience* **10**, 1728–1744 (1990).

[44] Scala, F. *et al.* Phenotypic variation of transcriptomic cell types in mouse motor cortex. *Nature* **598**, 144–150 (2021).

[45] Ascoli, G. A., Donohue, D. E. & Halavi, M. Neuromorpho. org: a central resource for neuronal morphologies. *Journal of Neuroscience* **27**, 9247–9251 (2007).

[46] Vogels, T. P. & Abbott, L. F. Signal propagation and logic gating in networks of integrate-and-fire neurons. *Journal of neuroscience* **25**, 10786–10795 (2005).

[47] Burkitt, A. N. A review of the integrate-and-fire neuron model: I. homogeneous synaptic input. *Biological cybernetics* **95**, 1–19 (2006).

[48] Rall, W. Distinguishing theoretical synaptic potentials computed for different soma-dendritic distributions of synaptic input. *Journal of neurophysiology* **30**, 1138–1168 (1967).

[49] Inagaki, H. K., Inagaki, M., Romani, S. & Svoboda, K. Low-dimensional and monotonic preparatory activity in mouse anterior lateral motor cortex. *Journal of Neuroscience* **38**, 4163–4185 (2018).

[50] Lebovich, L., Darshan, R., Lavi, Y., Hansel, D. & Loewenstein, Y. Idiosyncratic choice bias naturally emerges from intrinsic stochasticity in neuronal dynamics. *Nature human behaviour* **3**, 1190–1202 (2019).

[51] van Vreeswijk, C. & Sompolinsky, H. Chaotic balanced state in a model of cortical circuits. *Neural computation* **10**, 1321–1371 (1998).

[52] Kim, E., Bari, B. A. & Cohen, J. Y. Subthreshold basis for reward-predictive persistent activity in mouse prefrontal cortex. *Cell reports* **35**, 109082 (2021).

[53] DeWeese, M. R. & Zador, A. M. Non-gaussian membrane potential dynamics imply sparse, synchronous activity in auditory cortex. *Journal of Neuroscience* **26**, 12206–12218 (2006).

[54] Kiritani, T., Pala, A., Gasselin, C., Crochet, S. & Petersen, C. C. Membrane potential dynamics of excitatory and inhibitory neurons in mouse barrel cortex during active whisker sensing. *Plos one* **18**, e0287174 (2023).

[55] Gabernet, L., Jadhav, S. P., Feldman, D. E., Carandini, M. & Scanziani, M. Somatosensory integration controlled by dynamic thalamocortical feed-forward inhibition. *Neuron* **48**, 315–327 (2005).

[56] Cruikshank, S. J., Lewis, T. J. & Connors, B. W. Synaptic basis for intense thalamocortical activation of feedforward inhibitory cells in neocortex. *Nature neuroscience* **10**, 462–468 (2007).

[57] Landau, I. D., Egger, R., Dercksen, V. J., Oberlaender, M. & Sompolinsky, H. The impact of structural heterogeneity on excitation-inhibition balance in cortical networks. *Neuron* **92**, 1106–1121 (2016).

[58] Nevian, T., Larkum, M. E., Polsky, A. & Schiller, J. Properties of basal dendrites of layer 5 pyramidal neurons: a direct patch-clamp recording study. *Nature neuroscience* **10**, 206–214 (2007).

[59] Larkum, M. E., Nevian, T., Sandler, M., Polsky, A. & Schiller, J. Synaptic integration in tuft dendrites of layer 5 pyramidal neurons: a new unifying principle. *Science* **325**, 756–760 (2009).

[60] Larkum, M. E., Zhu, J. J. & Sakmann, B. A new cellular mechanism for coupling inputs arriving at different cortical layers. *Nature* **398**, 338–341 (1999).

[61] Waters, J., Larkum, M., Sakmann, B. & Helmchen, F. Supralinear ca2+ influx into dendritic tufts of layer 2/3 neocortical pyramidal neurons in vitro and in vivo. *Journal of Neuroscience* **23**, 8558–8567 (2003).

[62] Li, B., Routh, B. N., Johnston, D., Seidemann, E. & Priebe, N. J. Voltage-gated intrinsic conductances shape the input-output relationship of cortical neurons in behaving primate v1. *Neuron* **107**, 185–196 (2020).

[63] Amsalem, O., Van Geit, W., Muller, E., Markram, H. & Segev, I. From neuron biophysics to orientation selectivity in electrically coupled networks of neocortical l2/3 large basket cells. *Cerebral Cortex* **26**, 3655–3668 (2016).

[64] Major, G., Larkum, M. E. & Schiller, J. Active properties of neocortical pyramidal neuron dendrites. *Annual review of neuroscience* **36**, 1–24 (2013).

[65] Palmer, L. M. *et al.* Nmda spikes enhance action potential generation during sensory input. *Nature neuroscience* **17**, 383–390 (2014).

[66] Litwin-Kumar, A., Rosenbaum, R. & Doiron, B. Inhibitory stabilization and visual coding in cortical circuits with multiple interneuron subtypes. *Journal of neurophysiology* **115**, 1399–1409 (2016).

[67] Palmigiano, A. *et al.* Structure and variability of optogenetic responses identify the operating regime of cortex. *bioRxiv* 2020–11 (2021).

[68] Yamashita, T. *et al.* Membrane potential dynamics of neocortical projection neurons driving target-specific signals. *Neuron* **80**, 1477–1490 (2013).

[69] Gentet, L. J., Avermann, M., Matyas, F., Staiger, J. F. & Petersen, C. C. Membrane potential dynamics of gabaergic neurons in the barrel cortex of behaving mice. *Neuron* **65**, 422–435 (2010).

[70] Tasic, B. *et al.* Shared and distinct transcriptomic cell types across neocortical areas. *Nature* **563**, 72–78 (2018).

[71] Poulet, J. F. & Petersen, C. C. Internal brain state regulates membrane potential synchrony in barrel cortex of behaving mice. *Nature* **454**, 881–885 (2008).

[72] Rubin, R., Abbott, L. & Sompolinsky, H. Balanced excitation and inhibition are required for high-capacity, noise-robust neuronal selectivity. *Proceedings of the National Academy of Sciences* **114**, E9366–E9375 (2017).

[73] Darshan, R., Wood, W., Peters, S., Leblois, A. & Hansel, D. A canonical neural mechanism for behavioral variability. *Nature communications* **8**, 15415 (2017).

[74] Abdelfattah, A. S. *et al.* Bright and photostable chemigenetic indicators for extended in vivo voltage imaging. *Science* **365**, 699–704 (2019).

[75] Adam, Y. *et al.* Voltage imaging and optogenetics reveal behaviour-dependent changes in hippocampal dynamics. *Nature* **569**, 413–417 (2019).

[76] Carnevale, N. T. & Hines, M. L. *The NEURON book* (Cambridge University Press, 2006).

[77] Harris, C. R. *et al.* Array programming with numpy. *Nature* **585**, 357–362 (2020).

[78] Hunter, J. D. Matplotlib: A 2d graphics environment. *Computing in science & engineering* **9**, 90–95 (2007).

[79] Yu, J., Hu, H., Agmon, A. & Svoboda, K. Recruitment of gabaergic interneurons in the barrel cortex during active tactile behavior. *Neuron* **104**, 412–427 (2019).

[80] Scala, F. *et al.* Layer 4 of mouse neocortex differs in cell types and circuit organization between sensory areas. *Nature communications* **10**, 1–12 (2019).

[81] MacLean, J. N., Watson, B. O., Aaron, G. B. & Yuste, R. Internal dynamics determine the cortical response to thalamic stimulation. *Neuron* **48**, 811–823 (2005).

[82] Oswald, M. J., Tantirigama, M. L., Sonntag, I., Hughes, S. M. & Empson, R. M. Diversity of layer 5 projection neurons in the mouse motor cortex. *Frontiers in cellular neuroscience* **7**, 174 (2013).

[83] Lefort, S., Tomm, C., Sarria, J.-C. F. & Petersen, C. C. The excitatory neuronal network of the c2 barrel column in mouse primary somatosensory cortex. *Neuron* **61**, 301–316 (2009).

[84] Dougherty, S. *et al.* Mice lacking the transcriptional coactivator pgc-1α exhibit alterations in inhibitory synaptic transmission in the motor cortex. *Neuroscience* **271**, 137–148 (2014).

[85] Bindman, L., Meyer, T. & Prince, C. Comparison of the electrical properties of neocortical neurones in slices in vitro and in the anaesthetized rat. *Experimental brain research* **69**, 489–496 (1988).

[86] Paré, D., Shink, E., Gaudreau, H., Destexhe, A. & Lang, E. J. Impact of spontaneous synaptic activity on the resting properties of cat neocortical pyramidal neurons in vivo. *Journal of neurophysiology* **79**, 1450–1460 (1998).

[87] Amsalem, O. *et al.* An efficient analytical reduction of detailed nonlinear neuron models. *Nature communications* **11**, 1–13 (2020).

[88] Argaman, T. & Golomb, D. Does layer 4 in the barrel cortex function as a balanced circuit when responding to whisker movements? *Neuroscience* **368**, 29–45 (2018).

[89] Rubin, D. B., Van Hooser, S. D. & Miller, K. D. The stabilized supralinear network: a unifying circuit motif underlying multi-input integration in sensory cortex. *Neuron* **85**, 402–417 (2015).

[90] Ahmadian, Y., Rubin, D. B. & Miller, K. D. Analysis of the stabilized supralinear network. *Neural computation* **25**, 1994–2037 (2013).

[91] Curtis, J. C. & Kleinfeld, D. Phase-to-rate transformations encode touch in cortical neurons of a scanning sensorimotor system. *Nature neuroscience* **12**, 492–501 (2009).

[92] Crochet, S., Poulet, J. F., Kremer, Y. & Petersen, C. C. Synaptic mechanisms underlying sparse coding of active touch. *Neuron* **69**, 1160–1175 (2011).

[93] Sachidhanandam, S., Sreenivasan, V., Kyriakatos, A., Kremer, Y. & Petersen, C. C. Membrane potential correlates of sensory perception in mouse barrel cortex. *Nature neuroscience* **16**, 1671–1677 (2013).

[94] Yang, H., Kwon, S. E., Severson, K. S. & O'connor, D. H. Origins of choice-related activity in mouse somatosensory cortex. *Nature neuroscience* **19**, 127–134 (2016).

[95] Isett, B. R., Feasel, S. H., Lane, M. A. & Feldman, D. E. Slip-based coding of local shape and texture in mouse s1. *Neuron* **97**, 418–433 (2018).

[96] Hansel, D. & Van Vreeswijk, C. How noise contributes to contrast invariance of orientation tuning in cat visual cortex. *Journal of Neuroscience* **22**, 5118–5128 (2002).
